# Snapshot of a Child and Adolescent Psychiatric ER during Pandemic

**DOI:** 10.1192/j.eurpsy.2022.1085

**Published:** 2022-09-01

**Authors:** F. Arain, A. Tohid, M. Jawad, A. Rashid, P. Korenis, J. Sanchez-Lacay

**Affiliations:** 1 BronxCare Health System Icahn School of Medicine at Mount Sinai, Child & Adolescent Psychiatry, Bronx, United States of America; 2 University of Southern California, Psychiatry, Los Angeles, United States of America; 3 King Edward Medical University, Psychiatry, Lahore, Pakistan; 4 University of Southern California, Psychiatry, Bronx, United States of America

## Abstract

**Introduction:**

The COVID-19 pandemic has disrupted numerous fundamental systems ranging from businesses to education system. The long-term consequences of the Pandemic, namely virtual learning and prolonged social isolation are coming to fruition in Child/Adolescent-Psychiatric Emergency-Rooms (CAP-ER). Discontinuity of in-person attendance of schools has poorly impacted the mental health of children and adolescents (C&A) of low-socioeconomic areas, who often rely on schools for meals, physical activity, and mental-health support. An increase in agitation, suicidal ideation, and a declining school performance has been observed in such situations.

**Objectives:**

The primary objective of this study is to explore the increase in these symptoms as the presenting complaint in the psychiatric ER.

**Methods:**

Between April to June 2021 a cross-sectional quality improvement (QI) study was done on children presenting to CAP-ER BronxCare-Hospital NY with psychiatric complaints. Concomitant substance use disorder was determined using CRAFT questionnaire.

**Results:**

Our data comprised 209 patients (84 M/125 F) with 79 children and 130 adolescents. Ethnicity: 116 Hispanics (56%), 84 African Americans (40%), and 9 others. The most common presenting complaints were aggression (111, 53%), suicidal ideation/suicide attempt (50, 24%), acute exacerbation of chronic illness (7, 3.3%), accidental overdosage (5, 2.3%) and others (36, 17.4). Marijuana was the most used substance (34 patients).

**Conclusions:**

There has been a surge in severity of presentation of psychiatric disorders among children and adolescents, aggression so far, the most prevalent. Further studies are needed to delineate the social links with this high emergent load and pandemic.

**Disclosure:**

No significant relationships.

